# Economic crisis and increased immigrant mobility: new challenges in managing Chagas disease in Europe

**DOI:** 10.2471/BLT.13.134072

**Published:** 2014-09-09

**Authors:** Yves Jackson, Monica Varcher Herrera, Joaquim Gascon

**Affiliations:** a Department of Community Medicine, Primary Care and Emergency Medicine, Geneva University Hospitals, rue Gabrielle-Perret-Gentil 6, 1211 Geneva 14, Switzerland.; b Barcelona Centre for International Health Research, ISGLOBAL, Barcelona, Spain.

In Europe, there is evidence of the effect of the global financial crisis and austerity measures on vulnerable people’s health: increasing risky health behaviours, difficulties accessing health care and poorer mental, maternal and child health.^1^ There are little data on the extent to which communicable diseases are affected by the economic crisis in developed countries. However, a meta-analysis suggests worse health outcomes affecting the whole population, with infants, elderly, poor and/or homeless people, prisoners and immigrants carrying the main burden.^2^

Europe has a substantial burden of infectious diseases in vulnerable groups with social and economic disadvantages. In the past two decades this region has received more than 3.5 million Latin American immigrants, with a predominance of young-to-middle aged women, many of whom are undocumented.^3^ Chagas disease, a potential life-threating parasitic infection endemic in Latin America, has recently emerged in Europe, affecting an estimated 80 000 to 120 000 Latin American immigrants. In European countries, Chagas disease costs 16 million United States dollars (US$) for health care and US$ 137 million as a result of lost productivity annually.^4^

Chagas disease transmission through congenital and unscreened blood and organ donations has been identified in several European countries. Italy and Spain, two countries severely hit by the economic crisis, host the largest Latin American communities at risk and have reported the highest number of Chagas cases.^5^ Undetected and untreated, 20–40% of infected people develop severe cardiac, digestive or neurologic complications requiring complex, costly and long-term medical attention.

Despite evidence of the economic and health benefits of introducing targeted screening and treatment strategies among risk groups, Chagas disease-related health needs remain largely unmet in Europe. It is estimated that 90% of infected persons living in Europe have yet to be identified.^5^ The recommended transmission-control strategies include screening pregnant women, their babies and other family members, and testing blood and organ donors at risk. But the identification of infected individuals is particularly hindered by the long clinically-silent phase, the poor specificity and high diversity of possible symptoms, the need for specific diagnostic procedures and the lack of awareness among health professionals. Moreover, infected immigrants at risk in Europe often face administrative and economic barriers preventing them from obtaining access to care.

Europe’s current economic crisis and subsequent austerity measures have been significantly affecting the management of Chagas disease. Since 2008, several European countries have been cutting health and social services for vulnerable immigrants. Spain, for example, issued a Royal Decree in 2012 severely restricting access to preventive and primary health care for undocumented immigrants.

As a result of the decreasing economic opportunities in the countries most affected – notably Italy, Portugal and Spain – immigrants have been moving to other European countries or going back to their countries of origin.^3^ Immigrant families have in some instances been forced to split, with male and female members targeting specific job opportunities in different places. This jeopardizes continuity of care and challenges the recommended screening of the whole family after a case of infection has been identified.

While most infected immigrants initially lived in Spain – where effective responses to Chagas disease have been developed over the last decade – some immigrants have recently moved to other European countries, with few or no Chagas-disease programmes, thus raising the likelihood of further transmission.

Therefore, there is a need for European countries to implement evidence-based public health and clinical interventions to optimize Chagas-disease management and control in Europe in the context of current migration patterns.

The community at highest risk is that of the Bolivians who accounted for the majority of the 4290 cases identified in Europe up to 2009.^5^ Around 300 000 Bolivians live in Europe, frequently clustered in closely knit communities.^3^ Where the implementation of large-scale programmes targeting all Latin American immigrants at risk is not feasible, efforts should instead focus on the Bolivian community. While this may pose the risk of missing some cases from other communities, it would provide acceptable effectiveness. Therefore, all efforts should be made to identify areas where recently arrived Bolivian immigrants have settled and to develop preventive and curative Chagas-disease programmes targeting these communities while taking care to avoid any risk of discrimination or human rights’ abuse.

While developing specialized vertical Chagas-disease programmes might entail major financial and organizational constraints, integrating them into existing primary health care programmes targeting immigrants is likely to be the most effective strategy.

Improving awareness, ensuring that diagnostic facilities are available and developing capacities to orientate patients towards the best treatment options within such settings (including paediatric and gynaecology and obstetrics facilities) would help identify cases early and reduce the risk of transmission.

Primary care represents the optimal setting for providing an effective and global approach to the whole family. Close collaboration between primary and specialized care services has proven beneficial for fostering the optimal conditions to provide treatment in the safest conditions.

Chagas-disease patients frequently present concomitant noncommunicable diseases, requiring regular contact with primary-care services, highlighting the need for comprehensive, primary care-based patient management.

Strong coordination and effective communication between health institutions providing services for immigrants across Europe would allow for better continuity of care as immigrants move. Benefits include reducing the risk of loss to follow-up of newborns undergoing the months-long multi-stage diagnostic procedure to identify congenital transmission from infected mothers and improving the post-treatment clinical follow-up of all cases.

Chagas disease is deeply embedded in social, economic and cultural factors, which also affect immigrant communities’ health behaviour. Immigrants might not engage with programmes for different reasons. These could include lack of awareness, fear of stigma and deportation, or negative messages about the disease. In recent years, organizations of Chagas-disease patients have sprung up in Latin America, North America and Europe. As a part of the international federation of associations of people affected by Chagas disease (FINDECHAGAS, http://www.findechagas.com), they have been effective in raising awareness, spreading information and facilitating contacts between communities and health systems in areas with Chagas disease burden. Strengthening community-based intervention with the support of immigrants’ organizations may therefore harness benefits in terms of effectiveness of screening and transmission-control programmes in communities that are often hard to reach.

As few European countries have public health policies on Chagas-disease control, this leads to persisting transmission. Considering congenital infection as the main route of transmission outside endemic areas, the high proportion of female Latin American immigrants of child-bearing age and the proven favourable cost–effect ratio of screening,^6^ priority attention should be paid to identifying infected pregnant women in areas hosting communities at risk. Adding Chagas-disease serology to other prenatal blood tests in well-defined populations would incur extra costs, but would harness major public health and individual benefits.

Safe blood transfusion should be implemented in countries hosting populations at risk. This can be done by excluding donors after the identification of Chagas disease risks or by discarding infected donations following universal blood testing. By carefully selecting the criteria for screening (for instance Bolivian origin or long-term residency in rural area of endemic countries, history of unscreened blood transfusion in Latin America or Chagas disease in the mother), a single or a limited number of reference centres would be sufficient to process all samples at little extra cost.

Economic factors may reduce public health and clinical capacities to address Chagas disease and increase immigrants’ vulnerability. However, the experience gained during the past decade in Europe in Chagas disease management can be used to design optimal control strategies in newly-affected countries.
